# Hemophagocytic lymphohistiocytosis induced by nivolumab/ipilimumab combination therapy: A case of lung adenocarcinoma that responded to early steroid pulse therapy

**DOI:** 10.1002/cnr2.1960

**Published:** 2024-01-09

**Authors:** Satoru Hagiwara, Junko Tanizaki, Hidetoshi Hayashi, Yoriaki Komeda, Naoshi Nishida, Akihiro Yoshida, Tomoki Yamamoto, Takuya Matsubara, Masatoshi Kudo

**Affiliations:** ^1^ Department of Gastroenterology and Hepatology Kindai University Faculty of Medicine Osaka Japan; ^2^ Department of Medical Oncology Kindai University Faculty of Medicine Osaka Japan

**Keywords:** hemophagocytic lymphohistiocytosis, immune checkpoint inhibitors, lung carcinoma, steroid pulse therapy

## Abstract

**Background:**

Immune checkpoint inhibitors have been reported to have excellent therapeutic effects on various malignant tumors. However, immune‐related adverse events can occur, targeting various organs.

**Case presentation:**

A 49‐year‐old male with lung carcinoma was started on carboplatin + pemetrexed + nivolumab (every 3 weeks) + ipilimumab (every 6 weeks), and nivolumab/ipilimumab was administered in the 3rd course. Subsequently, fever and fatigue developed, and grade 3 liver damage was also noted, so he was admitted to Kindai University Hospital. A bone marrow aspirate examination was performed on the third day of illness, and a definitive diagnosis of hemophagocytic lymphohistiocytosis (HLH) was made. It was determined that immediate therapeutic intervention was necessary, and pulse therapy with methylprednisolone was started on the third day of illness. After 3 days of pulse treatment, a rapid recovery of platelet values, a decrease in ferritin levels, and a decrease in lactate dehydrogenase were observed. Subjective symptoms such as fever and fatigue also quickly improved.

**Conclusion:**

Early diagnosis and treatment for HLH resulted in a positive response. The number of HLH cases may increase in the future due to the expansion of immune checkpoint inhibitor indications.

## 1 INTRODUCTION

Anti‐PD‐1 antibodies, which are immune checkpoint inhibitors (ICIs), inhibit the binding of PD‐1 and PD‐L1, mainly maintaining T‐cell activation and exerting anti‐tumor effects.^1^ Anti‐CTLA‐4 antibodies also act to activate antigen presentation by inhibiting the binding of CTLA‐4 molecules on T cells to B7 on antigen‐presenting cells.^2^, ^3^ Nivolumab and low‐dose ipilimumab, in combination with chemotherapy (carboplatin/pemetrexed [CBDCA/PEM]: 2 cycles), have become one of the standard first‐line treatments for advanced non‐small cell lung cancer.^4^ ICIs have been reported to have excellent therapeutic effects on many malignant tumors such as melanoma, renal cancer, gastric cancer, head and neck cancer, and lymphoma. On the other hand, immune‐related adverse events (irAEs) have become a problem and can occur targeting various organs.^5^, ^6^, ^7^ In particular, hemophagocytic lymphohistiocytosis (HLH) due to ICI is rare among irAEs, and many aspects of its diagnosis and treatment remain unclear.^8^, ^9^, ^10^ We report a case of pulmonary adenocarcinoma in which early steroid pulse therapy was effective against hemophagocytic syndrome associated with nivolumab/ipilimumab combination therapy.

## 2 CASE PRESENTATION

A 49‐year‐old male presented with complaints of fever and fatigue. There was no relevant family history and the patient denied any drinking habit but mentioned smoking 10 cigarettes/day for 30 years.

In January 2021, the patient visited a nearby hospital due to difficulty in breathing; a large left pleural effusion was noted and thoracic drainage was performed. Signet cell carcinoma was detected in a cell block test using pleural fluid cytology, and after immunostaining (CK7 positive, CK20 negative), a diagnosis of lung adenocarcinoma was made. From March 2021, the 9LA regimen was selected and CBDCA + PEM + nivolumab (3 weeks apart) + ipilimumab (6 weeks apart) was started. Nivolumab/ipilimumab was administered in May 2021 as a stable disease response was observed at the end of the second course, but fever and malaise appeared in June 2021. When he visited Kindai University Hospital in the same month, it was discovered that he had grade 3 liver damage, and he was admitted for treatment.

The following findings were recorded at admission: height, 167 cm; weight, 56.0 kg; body temperature, 38.2°C; blood pressure, 100/70 mmHg; heart rate, 130 bpm; and respiratory rate, 18 breaths/min. The patient showed clear consciousness, no palpebral conjunctiva anemia, no bulbar conjunctival yellowing, and the abdomen was flat/soft, with a palpable liver and spleen. At admission, the patient was taking esomeprazole magnesium hydrate 10 mg/day, brotizolam 0.25 mg/day, and naproxen 100 mg/day.

**FIGURE 1:**
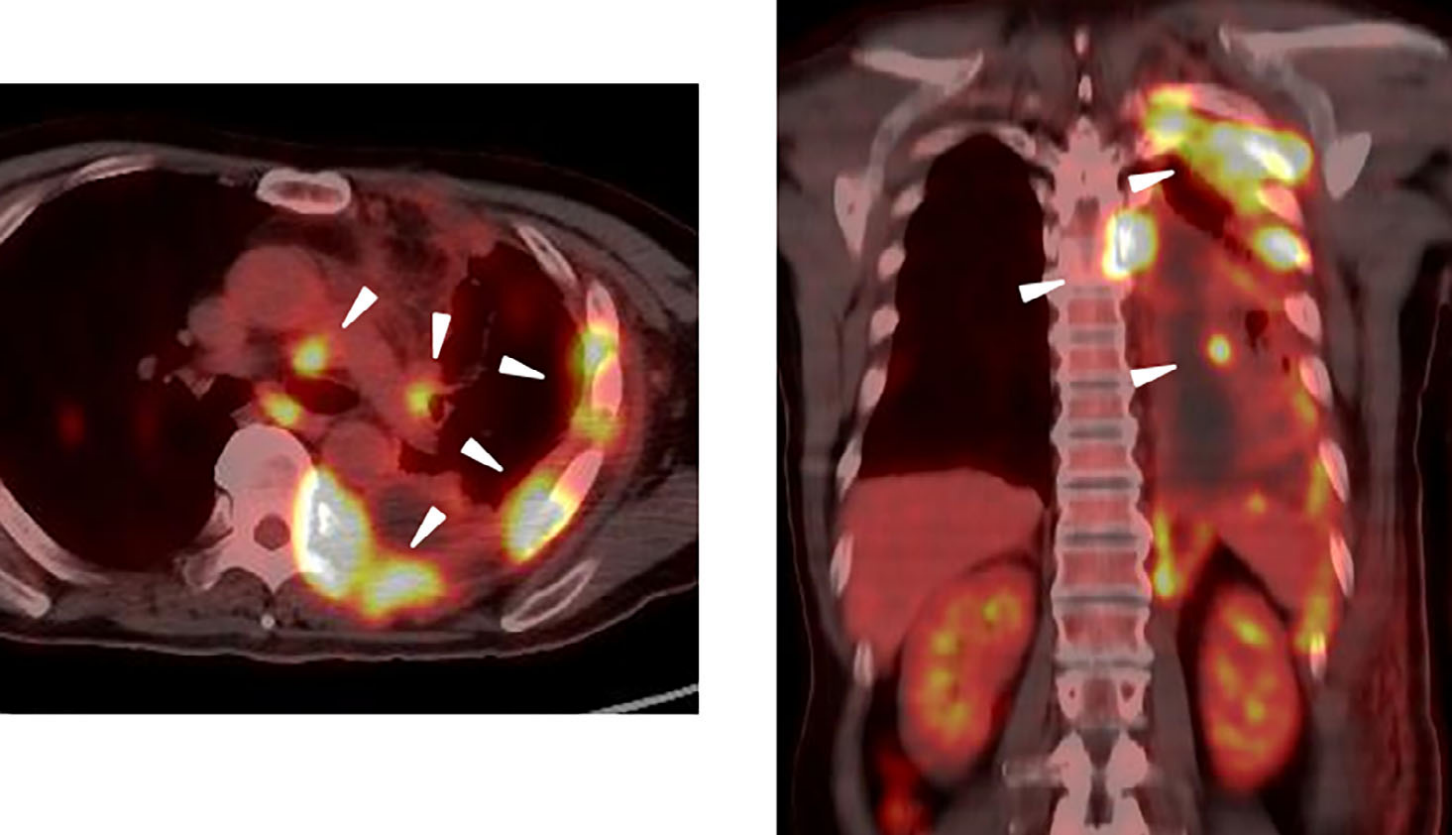
PET‐CT examination before Nivolumab/Ipilimumab combination therapy. Fluorodeoxyglucose accumulation was observed in the left pleura, lung field, and hilar lymph nodes. No obvious metastatic foci were noted in the liver.

**FIGURE 2:**
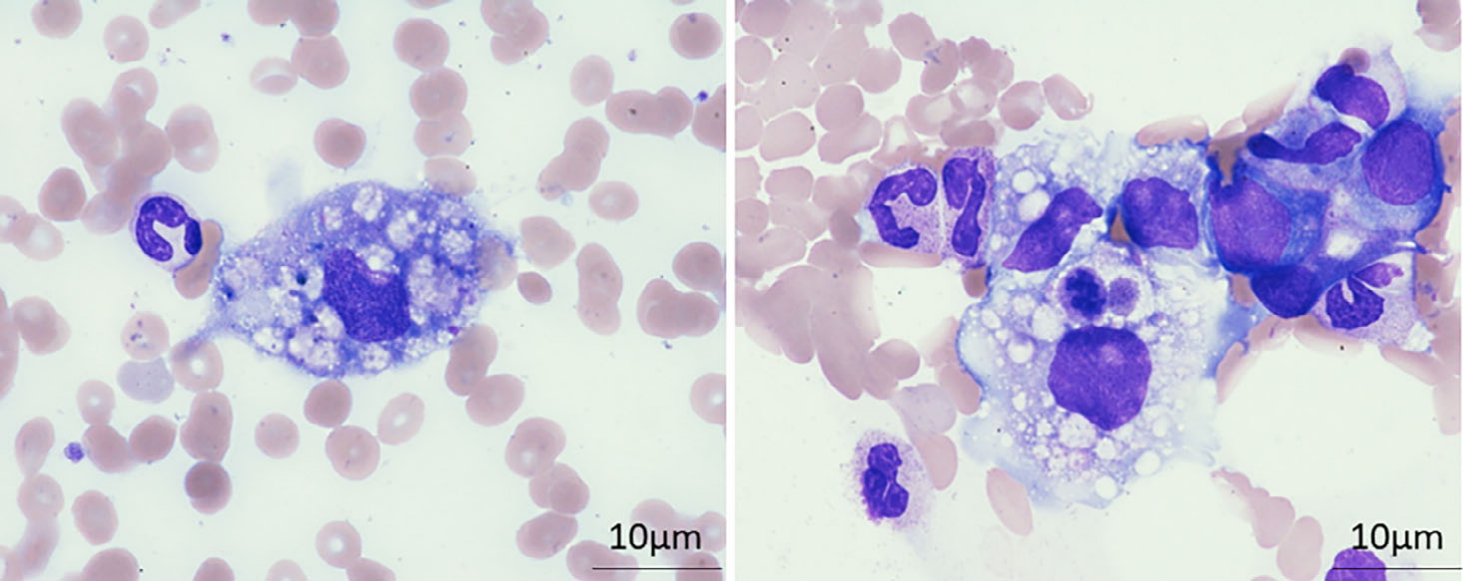
Bone marrow aspiration test. Multiple red blood cells, a small number of white blood cells, and macrophages that phagocytosed platelets were observed, consistent with hemophagocytic lymphohistiocytosis. Furthermore, no abnormal cells including lymphoma cells were observed in the smear. Bar is 10 μm.

Positron Emission Tomography ‐ Computed Tomography (PET‐CT) was performed as a baseline examination before nivolumab/ipilimumab combination therapy (Figure 1). Fluorodeoxyglucose accumulation was observed in the left pleura, lung field, and hilar lymph nodes. No obvious metastatic foci were noted in the liver. Based on the above, a diagnosis of cancerous pleurisy, multiple lung metastases, and multiple lymph node metastases was made. The results of blood testing at referral admission can be seen in Table 1. A marked increase in lactate dehydrogenase (LDH) and aspartate aminotransferase levels was observed, but the alanine aminotransferase increase was mild. A significant increase in the C‐reactive protein level was also observed. Hemophagocytosis was suspected due to the low hemoglobin and platelet levels and elevated triglyceride and LDH levels. Hepatitis virus markers were negative. A bone marrow aspirate examination was performed on the third day of illness. Multiple red blood cells, a small number of white blood cells, and macrophages that phagocytosed platelets were observed, consistent with HLH. Furthermore, no abnormal cells including lymphoma cells were observed in the smear (Figure 2).

**TABLE 1:** Laboratory data at admission.

Hematology		Blood chemistry		Viral markers	
WBC	3.69/μL (3.3–8.6)	TP	6.1 g/dL (6.6–8.1)	HBsAg	(−)
RBC	271 × 10^4^/μL (435–555 × 10^4^)	Albumin	1.8 g/dL (4.1–5.1)	HCVAb	(−)
Hemoglobin	7.6 g/dL (13.7–16.8)	BUN	17 mg/dL (8–20)	HA‐IgM	(−)
Hematocrit	23.3% (40.7–501)	Creatinine	0.56 mg/dL (0.65–1.07)	CMV‐IgM	(−)
Platelets	5.9 × 10^4^/μL (15.8–34.8 × 10^4^)	T‐Bil	2.6 mg/dL (0.4–1.5)	EBVVCA‐IgM	(−)
Neutrophils	70.7% (38–77)	D‐Bil	2.2 mg/dL (0–0.4)	Immunity	
Lymphocytes	18.2% (20.2–53.2)	ALP	8 U/L (38–113)	ANA	(−)
Eosinophils	0.3 (0.2–1.3)	Amylase	69 U/L (44–132)	AMA2	(−)
Endocrine		LDH	4186 U/L (124–222)	IgG	1793 mg/dL (861–1747)
ACTH	7.5 pg/mL (7.2–63.3)	AST	418 U/L (13–30)	IgM	57 mg/dL (33–183)
Cortisol	19.2 μg/dL (7.1–19.6)	ALT	102 U/L (10–42)	IgE	2434 IU/mL (0–232)
Coagulation		γGTP	257 U/L (13–64)	CRP	22.91 mg/dL (0–0.14)
PT	80.8% (70–130)	Triglycerides	321 mg/dL (40–150)	sIL‐2R	3477 U/mL (121–613)
INR	1.11	Ferritin	329 760 ng/mL (39.9–465)	PCT	0.82 ng/mL (0–0.5)

*Note*: Values in parenthesis is normal range.

Abbreviations: ACTH, adrenocorticotropic hormone; ALP, alkaline phosphatase; ALT, alanine aminotransferase; AMA2, anti‐mitochondrial antibody 2; ANA, antinuclear antibody; AST, aspartate aminotransferase; BUN, blood urea nitrogen; CMV, cytomegalovirus; CRP, C‐reactive protein; D‐Bil, direct bilirubin; EBVVCA, Epstein–Barr virus viral capsid antigen; HA, Hepatitis A; HBsAg, hepatitis B surface antigen; HCVAb, hepatitis C virus antibody; Ig, immunoglobulin; INR, international normalized ratio; LDH, lactate dehydrogenase; PCT, procalcitonin; PT, prothrombin time; RBC, red blood cells; sIL‐2R, soluble interleukin‐2 receptor; T‐Bil, total bilirubin; TP, total protein; WBC, white blood cells; γGTP, gamma‐glutamyl transpeptidase.

**FIGURE 3:**
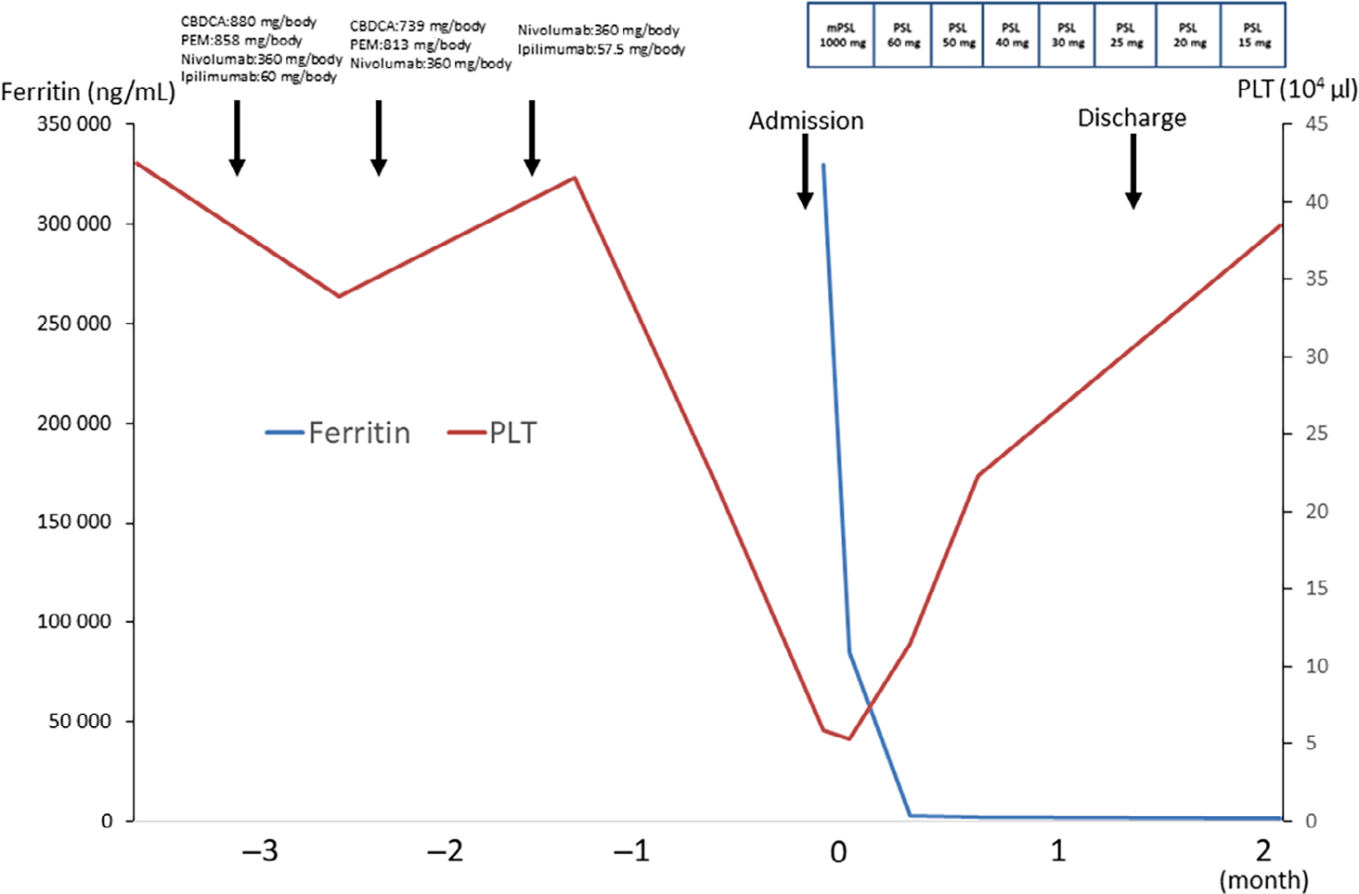
Clinical course of the patient. Fever and malaise developed after three courses of nivolumab/ipilimumab were administered for lung adenocarcinoma. Grade 3 liver damage was also noted, and the patient was hospitalized for treatment. After admission, pulse therapy with methylprednisolone (mPSL) was started on the third hospitalization day. After 3 days of pulse therapy, a rapid recovery of platelet values, a decrease in ferritin levels, and a decrease in lactate dehydrogenase were observed. Subjective symptoms such as fever and fatigue also quickly improved. The patient was discharged from hospital on the 35th day of illness, and prednisolone was gradually reduced to 15 mg/day, but the patient has not experienced any symptoms or recurrence of immune‐related hemophagocytic lymphohistiocytosis. CBDCA, carboplatin; PEM, pemetrexed.

**FIGURE 4:**
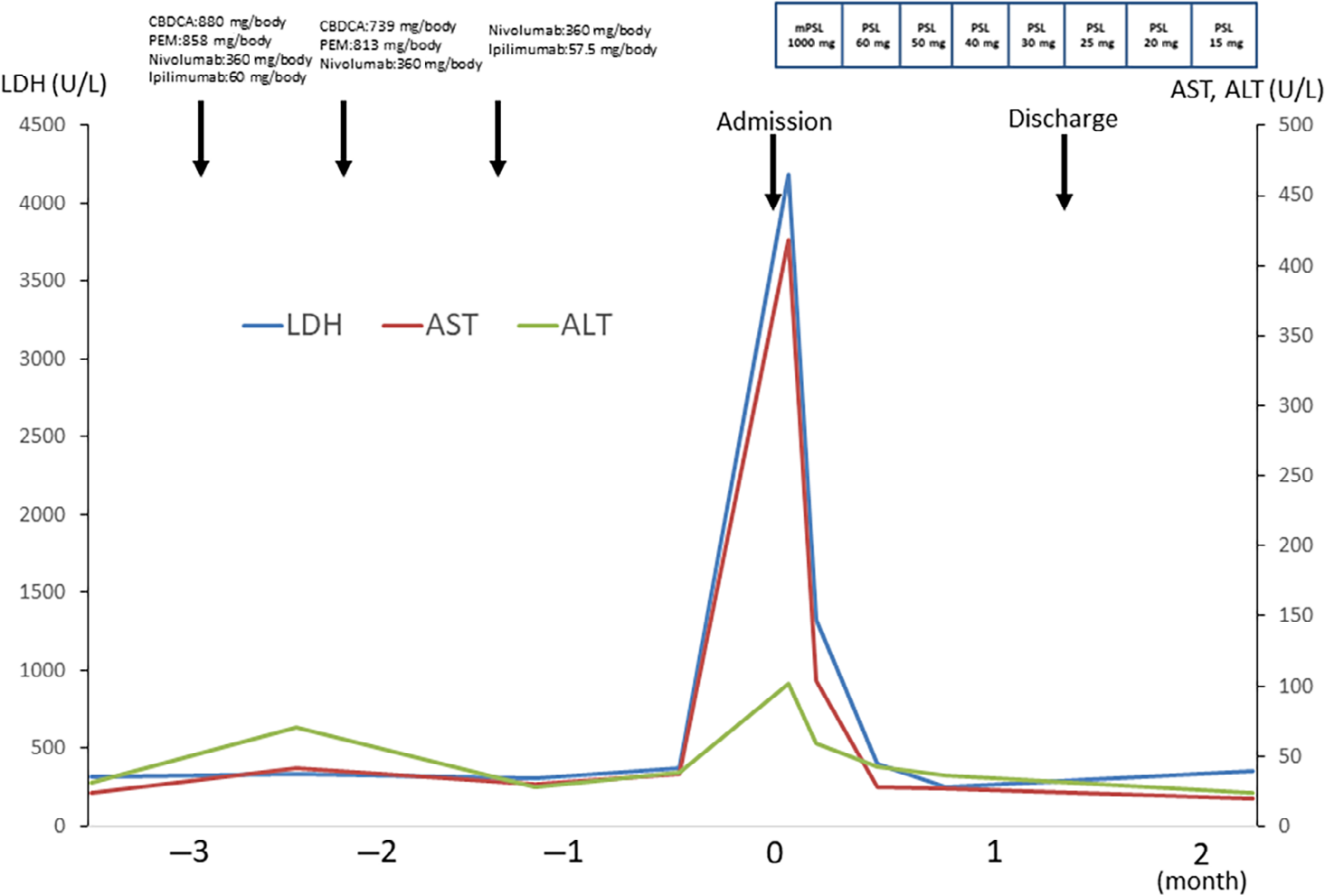
Clinical course of the patient. Fever and malaise developed after three courses of Nivolumab/Ipilimumab were administered for lung adenocarcinoma. Grade 3 liver damage was also noted, and the patient was hospitalized for treatment. After admission, pulse therapy with methylprednisolone (mPSL) was started on the third hospitalization day. After 3 days of pulse therapy, a rapid recovery of platelet values, a decrease in ferritin levels, and a decrease in lactate dehydrogenase were observed. Subjective symptoms such as fever and fatigue also quickly improved. The patient was discharged from hospital on the 35th day of illness, and prednisolone was gradually reduced to 15 mg/day, but the patient has not experienced any symptoms or recurrence of immune‐related hemophagocytic lymphohistiocytosis. ALT, alanine aminotransferase; AST, aspartate aminotransferase; CBDCA, carboplatin; LDH, lactate dehydrogenase; PEM, pemetrexed.

The clinical course after admission can be seen in Figures 3 and 4. Blood sampling performed on the day of hospitalization showed a decrease in platelets and an increase in LDH and triglyceride levels which led to the suspicion of hemophagocytosis, further confirmed through an additional test for serum ferritin, which revealed a remarkable value of 329 760 ng/mL. Based on these results, HLH was suspected, and a bone marrow aspiration test was performed on the third day of hospitalization to confirm the diagnosis. Bone marrow aspirate examination revealed macrophages in 20% of cases and clear signs of phagocytosis. It was determined that immediate therapeutic intervention was necessary, and pulse therapy with methylprednisolone (mPSL) was started on the same day. After 3 days of pulse treatment, a rapid recovery of platelet values, as well as decreased ferritin and LDH levels were observed. CRP and sIL2‐R also rapidly decreased. Subjective symptoms such as fever and fatigue also quickly improved. The patient was discharged from the hospital on the 35th day, and prednisolone was gradually reduced to 15 mg/day. The patient did not experience symptoms or recurrence of immune‐related HLH (irHLH) but died of respiratory failure due to progression of lung cancer.

## 3 DISCUSSION

HLH is a disease in which, in addition to a strong inflammatory response such as fever, activated macrophages and histiocytes in the reticuloendothelial tissue phagocytose the patient's own blood cells, resulting in marked cytopenia. There are primary (familial) and secondary causes, with the latter including infections, malignant tumors, and autoimmune diseases.^11^ A problem unique to ICIs is irAEs caused by autoimmune reactions. irAEs typically encompass endocrine disorders, skin eruptions, interstitial pneumonia, colitis, and liver damage,^5^, ^6^, ^7^ and can target all organs. A World Health Organization database analysis of cases of HLH after ICI administration, although infrequent, have been reported.^12^ Of the 49 883 cases in which ICIs were used for lung cancer, malignant melanoma, renal cancer, and other malignancies, some adverse events (AEs) occurred. HLH was observed in 38 of these cases of AEs (0.08%), and 10 of the 38 patients (26.3%) died. In four of these cases, HLH was the cause of death, highlighting its high mortality rate. Of the 38 patients, five received nivolumab/ipilimumab combination therapy. The median time from ICI administration to the onset of HLH was 6.7 weeks, but four cases (10.5%) developed the disease after the first ICI administration. In this case, HLH developed ca. 8 weeks after ICI administration, which constituted a more delayed onset.

HLH is generally reported as a subtype of cytokine storm syndrome.^13^, ^14^ Cytokine release syndrome (CRS) typically refers to the specific cytokine storm syndrome seen after chimeric antigen receptor‐T cell therapy characterized by very high interleukin (IL)‐6/CRP. Therefore, HLH after ICI administration is presumed to develop secondary to CRS. The results of a WHO database analysis regarding the onset of CRS due to ICI administration have also been reported,^15^ showing 58 cases of CRS in 80 700 patients (0.071%). Only two of the 58 patients with CRS died (3.4%), but the outcome was unknown in 20 cases (34.4%). The median time from ICI administration to CRS onset was 4 weeks. Two major pathogenic mechanisms for the onset of CRS after ICI are known. First, ICI administration activates T‐cells, which produce large amounts of interferon‐ɤ and tumor necrosis factor‐α. This activates innate immune cells such as macrophages and endothelial cells to produce IL‐1β, IL‐6, and tumor necrosis factor‐α, leading to CRS.^16^, ^17^ In the second pathway, cancer cell death due to T‐cell activation results in a large amount of damage‐associated molecular patterns, which increases cytokine production in innate immune cells and endothelial cells, leading to the onset of CRS.^18^ In any case, the onset of CRS after ICI administration can be said to be a preliminary state of irHLH, and early diagnosis and early treatment of CRS are considered important for improving the prognosis of irHLH.

Diagnosis of HLH during CRS, although related to chimeric antigen receptor T‐cell therapy, is described in the National Comprehensive Cancer Center Guidelines.^19^ These guidelines specify the criteria for considering the comorbidity of HLH during the onset of CRS. Elevated serum ferritin levels (>5000 ng/mL) and cytopenia are prerequisites. In addition, any of the following must be observed: a grade 3 or higher elevation in bilirubin, aspartate aminotransferase, alanine aminotransferase, or serum creatinine levels, or grade 3 or higher pulmonary edema. For the treatment of CRS, it is recommended to administer tocilizumab (8 mg/kg) in grade 2 cases, and dexamethasone (10 mg/body) in cases of treatment resistance. When CRS develops, fever, hypotension, tachycardia, and worsening of respiratory status occur, and the condition may rapidly worsen over the course of 1 h. Early diagnosis and treatment are therefore particularly important. In the Society for Immunotherapy of Cancer guidelines, there is an overlap of the pathology of CRS and HLH, and it has been specified that early treatment of CRS is important for improving HLH.^20^ It has also been reported that the pathophysiology of CRS and HLH after chimeric antigen receptor‐T therapy is different, and the prognosis is particularly poor for HLH.^21^ In this case, irHLH was suspected due to fever and a marked increase in serum ferritin, and a bone marrow aspiration test was performed on the same day, which confirmed the diagnosis of HLH. mPSL pulse therapy was started on the same day as the bone marrow aspiration examination. We believe that early diagnosis and treatment was a crucial factor in the successful management of this case. mPSL was effective in this case, but ruxolitinib and emapalumab have also been reported to be effective as therapeutic agents for HLH.^22^, ^23^, ^24^ Although selecting an appropriate treatment for ICI‐related HLH remains a future issue, we believe that the use of mPSL should be considered first.

On the other hand, nivolumab approved for the treatment of several cancers has been effective in Epstein–Barr virus (EBV)+ gastric cancer and NK/T lymphoma. Liu et al described retrospectively the outcome of seven patients treated with nivolumab for relapsing/refractory EBV‐associated HLH. All seven patients tolerated the treatment, and complete clinical response was achieved in five of them (72%). Nivolumab has the advantage of restoring the expression of HLH‐associated degranulation and costimulatory genes in CD8 T cells. Nivolumab can be considered only for EBV‐driven HLH, and further studies are needed to determine the efficacy and safety of nivolumab in EBV‐related HLH.^25^, ^26^

In conclusion, we experienced a case of lung adenocarcinoma in which early steroid pulse therapy was effective against hemophagocytic syndrome caused by nivolumab/ipilimumab combination therapy. The pathogenesis of CRS and HLH overlaps and early treatment of CRS is important for improving HLH.

## AUTHOR CONTRIBUTIONS

**Satoru Hagiwara:** Project administration; conceptualization (equal); data curation (equal). **Junko Tanizaki:** Conceptualization (equal); data curation (equal). **Hidetoshi Hayashi:** Conceptualization (equal); data curation (equal). **Yoriaki Komeda:** Conceptualization (equal); data curation (equal). **Naoshi Nishida:** Conceptualization (equal); data curation (equal). **Akihiro Yoshida:** Conceptualization (equal); data curation (equal). **Tomoki Yamamoto:** Conceptualization (equal); data curation (equal). **Takuya Matsubara:** Conceptualization (equal); data curation (equal). **Masatoshi Kudo:** Conceptualization (equal); project administration (equal).

## CONFLICT OF INTEREST STATEMENT

The authors have stated explicitly that there are no conflicts of interest in connection with this article.

## ETHICS STATEMENT

As this is a case report, there is no ethics committee certification.

## INFORMED CONSENT

Written consent was obtained from the patient to report the details of the case.

## Data Availability

Data included in article.
